# Genetic deletion of Abcc6 disturbs cholesterol homeostasis in mice

**DOI:** 10.1038/s41598-021-81573-1

**Published:** 2021-01-22

**Authors:** Bettina Ibold, Janina Tiemann, Isabel Faust, Uta Ceglarek, Julia Dittrich, Theo G. M. F. Gorgels, Arthur A. B. Bergen, Olivier Vanakker, Matthias Van Gils, Cornelius Knabbe, Doris Hendig

**Affiliations:** 1 Institut für Laboratoriums- und Transfusionsmedizin, Herz- und Diabeteszentrum Nordrhein-Westfalen, Universitätsklinik der Ruhr-Universität Bochum, 32545 Bad Oeynhausen, Germany; 2 Institut für Laboratoriumsmedizin, Klinische Chemie und Molekulare Diagnostik, Universitätsklinikum Leipzig, 04103 Leipzig, Germany; 3 University Eye Clinic Maastricht, Maastricht University Medical Center, 6202 AZ Maastricht, The Netherlands; 4 Netherlands Institute for Neurosciences (NIN-KNAW), Amsterdam, The Netherlands; 5 Academic Medical Centre, University of Amsterdam, 1100 DD Amsterdam, The Netherlands; 6 Center for Medical Genetics, Ghent University Hospital, 9000 Ghent, Belgium

**Keywords:** Biochemistry, Diseases, Medical research

## Abstract

Genetic studies link adenosine triphosphate-binding cassette transporter C6 (*ABCC6*) mutations to pseudoxanthoma elasticum (PXE). *ABCC6 s*equence variations are correlated with altered HDL cholesterol levels and an elevated risk of coronary artery diseases. However, the role of ABCC6 in cholesterol homeostasis is not widely known. Here, we report reduced serum cholesterol and phytosterol levels in Abcc6-deficient mice, indicating an impaired sterol absorption. Ratios of cholesterol precursors to cholesterol were increased, confirmed by upregulation of hepatic 3-hydroxy-3-methylglutaryl coenzyme A reductase (*Hmgcr*) expression, suggesting activation of cholesterol biosynthesis in *Abcc6*^*−/−*^ mice. We found that cholesterol depletion was accompanied by a substantial decrease in HDL cholesterol mediated by lowered ApoA-I and ApoA-II protein levels and not by inhibited lecithin-cholesterol transferase activity. Additionally, higher proprotein convertase subtilisin/kexin type 9 (Pcsk9) serum levels in *Abcc6*^*−/−*^ mice and PXE patients and elevated ApoB level in knockout mice were observed, suggesting a potentially altered very low-density lipoprotein synthesis. Our results underline the role of Abcc6 in cholesterol homeostasis and indicate impaired cholesterol metabolism as an important pathomechanism involved in PXE manifestation.

## Introduction

Mutations in the adenosine triphosphate-binding cassette transporter C6 (ABCC6) gene are responsible for pseudoxanthoma elasticum (PXE), a metabolic disease, hallmarked by a progressive elastic fiber calcification of the skin, eyes and cardiovascular system. Yellowish papules of the neck and flexure areas are often the first clinical signs of PXE, which were wrongly considered as xanthomas in early research^1^. Characteristic xanthomas are lesions in connective tissue formed by lipid-overloaded macrophages^2^. By contrast, skin lesions of PXE are due to morphological alterations of elastin fibers.

Recent studies verified that ABCC6 is involved in systemic pyrophosphate homeostasis although the physiological function and substrates of ABCC6 remain unknown/can be only presumed^3,4^. However, an in silico analysis from Hosen et al. showed that lipids and bile acids are particularly the most likely ABCC6 substrates^5^. Several ABCC6 *s*equence variations correlate with altered HDL cholesterol, triglyceride levels^6^, and an increased coronary risk^7^. A previous study demonstrated decreased HDL cholesterol plasma levels in 8-month-old *Abcc6*^*−/−*^ mice^8,9^. Furthermore, Guo et al. showed that a preventive treatment with atorvastatin inhibited calcifications in *Abcc6*^*−/−*^ mice^9^. However, the underlying mechanism why inhibition of 3-hydroxy-3-methylglutaryl coenzyme A reductase (HMGCR) can suppress ectopic mineralization is not clear^10^. Previously, we investigated cholesterol biosynthesis in dermal fibroblasts of PXE patients and observed increased HMGCR activity in comparison to healthy controls. Moreover, we showed increased proprotein convertase subtilisin/kexin type 9 (PCSK9) levels and altered ApoE expression^11^. Elastic fiber calcification observed in PXE is associated with decreased pyrophosphate levels in PXE patients and *Abcc6*^*−/−*^ mice^12,13^. Thus, bisphosphonates as nonhydrolyzable pyrophosphate analogues are potential candidates for treatment of PXE in addition to statins^13,14^. Bisphosphonates containing nitrogen inhibit alkaline phosphatase directly^15^, which degrades pyrophosphate and, therefore, promotes the mineralization process^3^. Moreover, both statins and bisphosphonates inhibit enzymes involved in the cholesterol biosynthesis^16^. Cholesterol has an important structural and metabolic function. It is an essential cell membrane component, a starting molecule for steroid hormone synthesis and for the formation of cofactors, vitamins and bile acids. But high blood cholesterol levels can have pathological consequences, for example, the development of vascular diseases. In addition, excessive accumulation of cholesterol is cell toxic and should be prevented or reduced via reverse cholesterol transport. Only hepatocytes and hormone-producing cells can metabolize cholesterol^17^. Maintenance of cholesterol homeostasis is primarily ensured by hepatic mechanisms, such as cholesterol biosynthesis, receptor-mediated uptake of lipoproteins, lipoprotein secretion, cholesterol degradation and storage (summarized in^18^). However, cells of peripheral tissues are capable of activating cholesterol synthesis due to their metabolic needs^19^.

Here, we used 6- and 12-month old *Abcc6*^*−/−*^ mice to reflect an early and a late disease stage of PXE to study the effects of an Abcc6 deficiency on cholesterol homeostasis. In this study, we found reduced HDL and total cholesterol levels in the serum of 12-month-old *Abcc6*^*−/−*^ mice. These *Abcc6*^*−/−*^ mice also showed consistently lower ApoA-I and ApoA-II levels and reduced phytosterol amounts in their serum. These results indicate that the ABC-transporter Abcc6 plays a relevant role in cholesterol metabolism and add new insights into the pathophysiology of PXE.

## Results

### Reduced serum lipid levels due to Abcc6 deficiency

**Figure 1:**
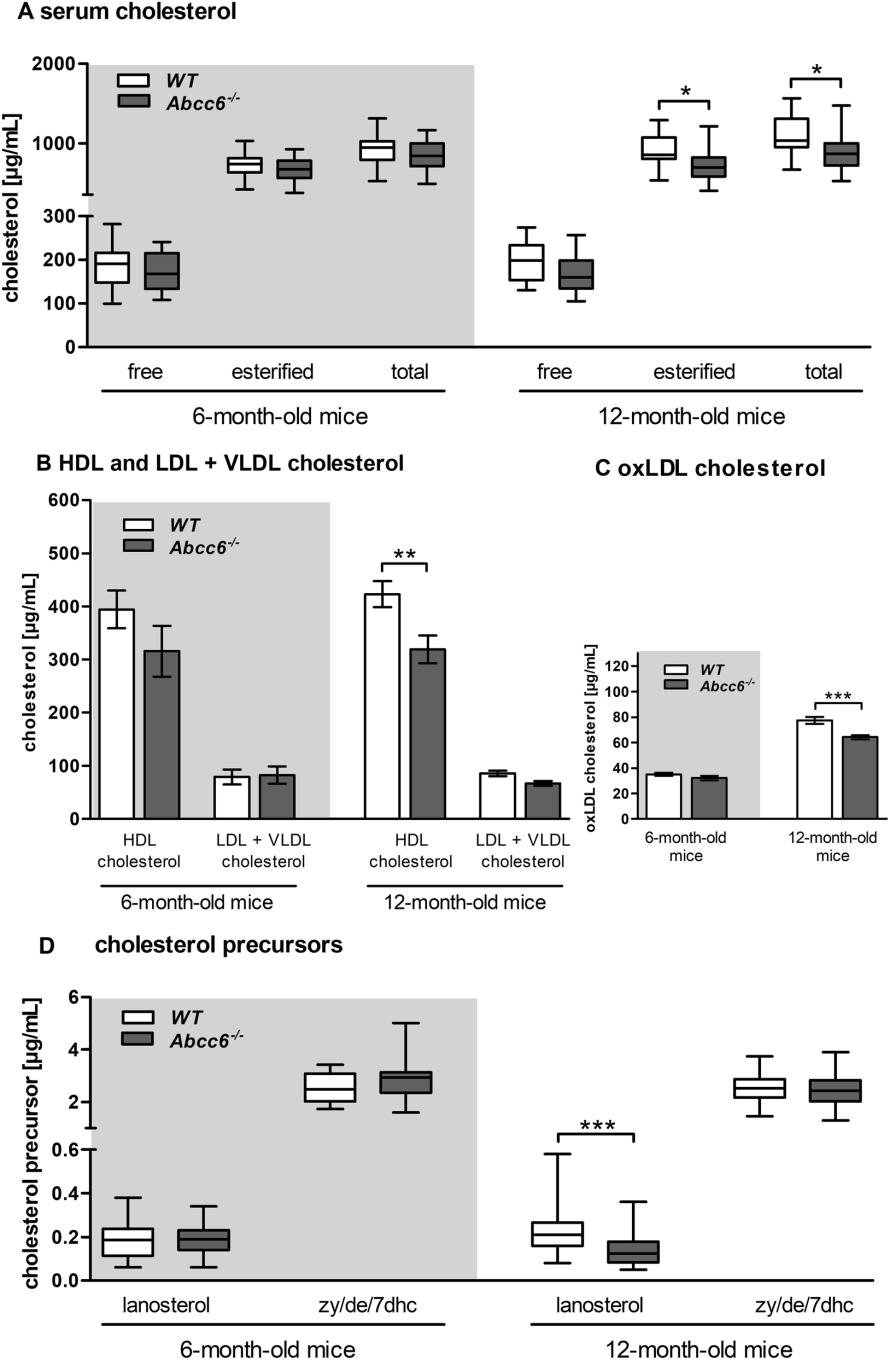
Reduced serum lipids in Abcc6 knockout mice. (**A**) Serum levels of free, esterified and total cholesterol of WT and *Abcc6*^*−/−*^ mice (6-month-old: WT n = 28, *Abcc6*^*−/−*^ n = 19; 12-month-old: WT n = 41, *Abcc6*^*−/−*^ n = 40). (**B**) Serum levels of HDL and LDL + VLDL cholesterol of WT and *Abcc6*^*−/−*^ mice (6-month-old: WT n = 8, *Abcc6*^*−/−*^ n = 4; 12-month-old: WT n = 18, *Abcc6*^*−/−*^ n = 17). (**C**) Quantification of oxLDL cholesterol serum levels of WT and *Abcc6*^*−/−*^ mice (6-month-old: WT n = 23, *Abcc6*^*−/−*^ n = 17; 12-month-old: WT n = 29, *Abcc6*^*−/−*^ n = 29). (**D**) Serum levels of free cholesterol precursors, lanosterol and zymosterol/desmosterol/7-dehydrocholesterol (zy/de/7dhc) of WT and *Abcc6*^*−/−*^ mice (6-month-old: WT n = 28, *Abcc6*^*−/−*^ n = 19; 12-month-old: WT n = 41, *Abcc6*^*−/−*^ n = 40). Data are mean ± SD; one factorial variance analysis (**A**,**B**,**D**) or Student’s *t* test (**C**); *p ≤ 0.05; **p ≤ 0.01; ***p ≤ 0.001.

In general no statistically significant differences were found between male and female mice. Free, esterified, and total serum cholesterol of 6-month-old *Abcc6*^*−/−*^ mice were not altered in comparison to WT mice. A significant decrease of esterified and total cholesterol is shown for 12-month-old *Abcc6*^*−/−*^ mice (Fig. 1A). We observed no reduction of HDL cholesterol in the serum of 6-month-old *Abcc6*^*−/−*^ mice (Fig. 1B), whereas significantly decreased levels of esterified and total serum cholesterol in 12-month-old *Abcc6*^*−/−*^ mice were attributed to a substantial decrease in HDL cholesterol (− 25%; Fig. 1B). A reduction of oxidized LDL (oxLDL) was solely detected in the serum of 12-month-old *Abcc6*^*−/−*^ mice (− 17%; Fig. 1C).

Comparing 6-month-old *Abcc6*^*−/−*^ mice and WT mice, alterations in serum concentrations of the cholesterol precursors lanosterol and zymosterol/desmosterol/7-dehydrocholesterol (zy/de/7dhc) were insignificant. However, lanosterol levels in serum of 12-month-old *Abcc6*^*−/−*^ mice were decreased by 38% (Fig. 1D). A significant reduction in the ratio of free lanosterol to cholesterol was found in 12-month-old *Abcc6*^*−/−*^ mice. By contrast, the ratio of free zy/de/7dhc to total cholesterol was significantly increased in both groups of *Abcc6*^*−/−*^ mice compared to WT mice (Table 1).

**Table 1:** Elevated cholesterol precursor to cholesterol ratios and reduction of relative plant sterol levels in Abcc6 knockout mice. Ratios of free cholesterol precursors [lanosterol and zymosterol/desmosterol/7-dehydrocholesterol (zy/de/7dhc)] and total phytosterols (brassicasterol, campesterol, β-sitosterol, stigmasterol) to total cholesterol (μg/mg ± SD) in serum of 6- and 12-month-old WT and Abcc6^*−/−*^ mice. Student’s *t* test; *p ≤ 0.05; **p ≤ 0.01; ***p ≤ 0.001.

Ratio (µg/mg)	6-month-old mice			12-month-old mice		
	WT (n = 28)	*Abcc6*^*−/−*^ (n = 19)	p-value	WT (n = 41)	*Abcc6*^*−/−*^ (n = 40)	p-value
Lanosterol_free_ to cholesterol_total_	0.21 ± 0.02	0.24 ± 0.02	0.233	0.21 ± 0.02	0.16 ± 0.01	0.005**
zy/de/dh_free_ to cholesterol_total_	0.51 ± 0.02	0.62 ± 0.03	0.001**	0.44 ± 0.02	0.53 ± 0.02	0.003**
Brassicasterol_total_ to cholesterol_total_	0.51 ± 0.02	0.62 ± 0.03	0.361	0.44 ± 0.02	0.53 ± 0.02	0.003**
Campesterol_total_ to cholesterol_total_	22.58 ± 0.89	18.42 ± 2.14	0.086	25.09 ± 0.93	19.18 ± 1.20	0.001***
β-Sitosterol_total_ to cholesterol_total_	8.84 ± 0.32	6.97 ± 0.97	0.109	8.89 ± 0.33	6.99 ± 0.47	0.002**
Stigmasterol_total_ to cholesterol_total_	0.95 ± 0.03	0.94 ± 0.10	0.863	1.11 ± 0.05	0.96 ± 0.05	0.038*

**Figure 2:**
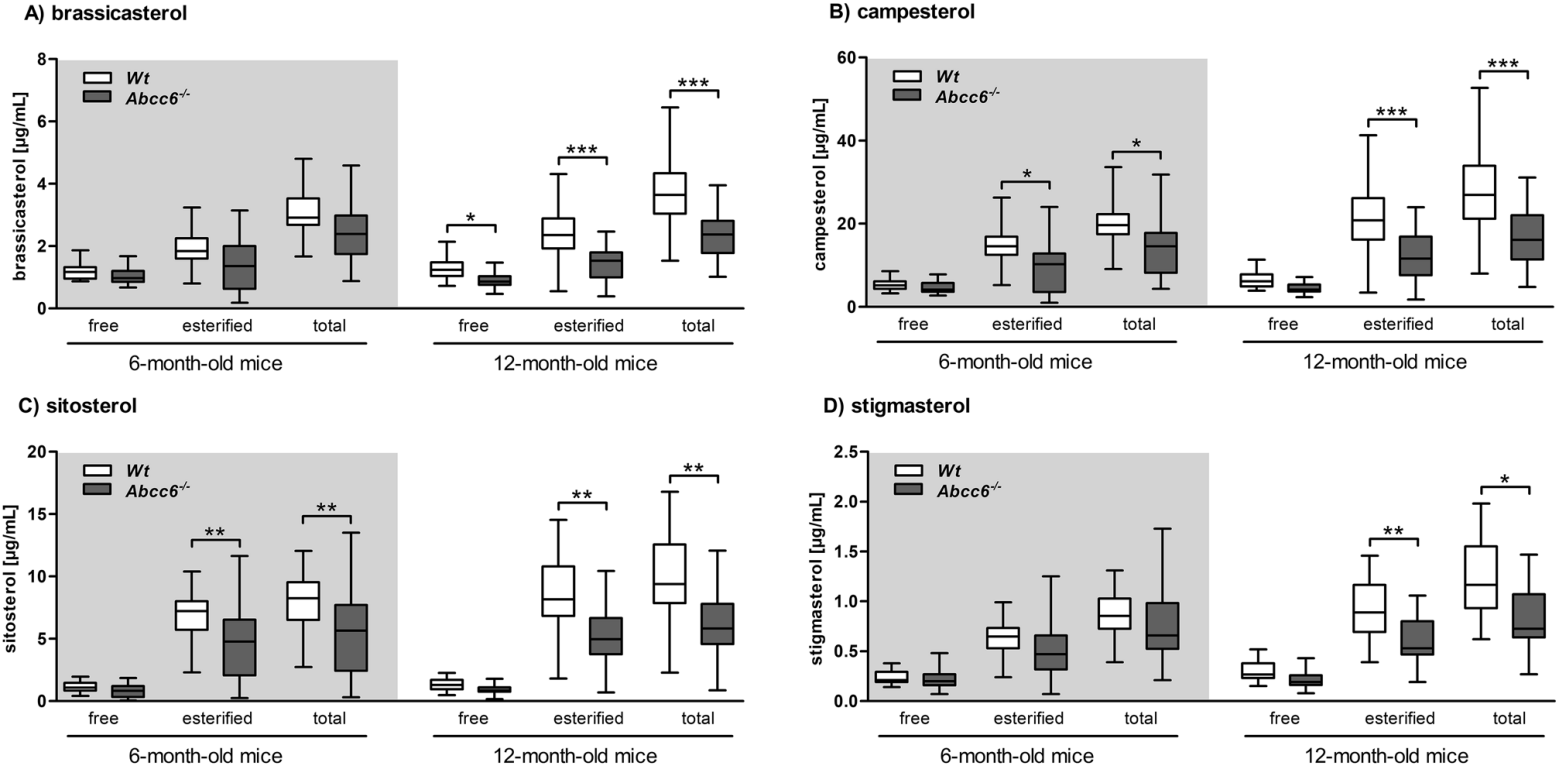
Reduced plant sterol levels in serum of Abcc6 knockout mice. Free, esterified and total phytosterol serum levels of WT and *Abcc6*^*−/−*^ mice (6-month-old: WT n = 28, *Abcc6*^*−/−*^ n = 19; 12-month-old: WT n = 41, *Abcc6*^*−/−*^ n = 40). (**A**) brassicasterol, (**B**) campesterol, (**C**) β-sitosterol and (**D**) stigmasterol. Data are mean ± SD; one factorial variance analysis; *p ≤ 0.05; **p ≤ 0.01; ***p ≤ 0.001.

We quantified lower serum plant sterol levels overall (free, esterified and total brassicasterol, campesterol, β-sitosterol and stigmasterol) of both groups of *Abcc6*^*−/−*^ mice analyzed in comparison to WT mice (Fig. 2A–D). We detected significant reductions of both esterified campesterol (6-month-old: − 32%; 12-month-old: − 45%) and total campesterol (6-month-old: − 27%; 12-month-old: − 41%) of 6- and 12-month-old *Abcc6* knockout mice (Fig. 2B). Esterified (6-month-old: − 30%; 12-month-old: − 40%) and total β-sitosterol (6-month-old: − 29%; 12-month-old: − 39%) were also significantly reduced in 6- and 12-month-old *Abcc6*^*−/−*^ mice (Fig. 2C). In addition, a significant reduction of free (− 41%), esterified (− 30%) and total brassicasterol (− 37%) was determined in the serum of 12-month-old *Abcc6*^*−/−*^ mice (Fig. 2A). Decreased serum levels of esterified (− 33%) and total stigmasterol (− 32%) were also identified in 12-month-old *Abcc6*^*−/−*^ mice (Fig. 2D). Table 1 summarizes that ratios of all phytosterols to cholesterol quantified were lower in both age groups of *Abcc6*^*−/−*^ mice compared to WT mice. The ratios of brassicasterol (− 0.6 µg/mg), campesterol (− 5.9 µg/mg), β-sitosterol (− 1.9 µg/mg), and stigmasterol (− 0.2 µg/mg) to cholesterol were significantly reduced in 12-month-old *Abcc6*^*−/−*^ mice (Table 1).

### Gene expression profile of key enzymes in cholesterol biosynthesis of liver tissue

**Figure 3:**
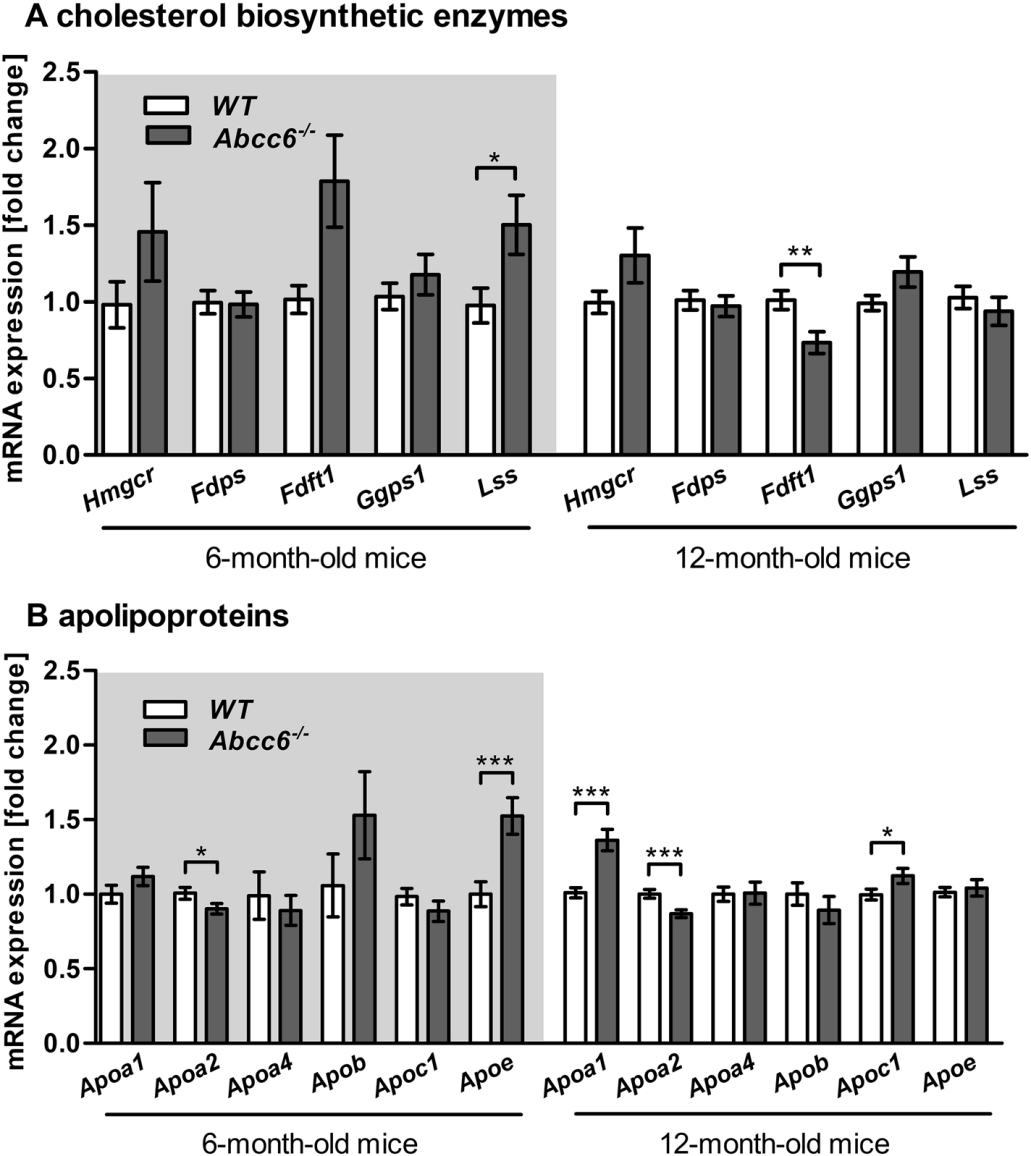
Altered gene expression of cholesterol biosynthetic enzymes and apolipoproteins in Abcc6 knockout mice. (**A**) Relative hepatic gene expression levels of cholesterol biosynthesis enzymes [3-hydroxy-3-methylglutaryl coenzyme A reductase (*Hmgcr*), farnesyl pyrophosphate synthase (*Fdps*), farnesyl-diphosphate farnesyltransferase 1 (*Fdft1*), geranylgeranyl pyrophosphate synthase 1 (*Ggps1*) and lanosterol synthase (*Lss*)] and (**B**) apolipoproteins (*Apoa1*, *Apoa2*, *Apoa4*, *Apob*, *Apoc1* and *Apoe*) of WT and *Abcc6*^*−/−*^ mice (6-month-old: WT n = 22, *Abcc6*^*−/−*^ n = 25; 12-month-old: WT n = 37, *Abcc6*^*−/−*^ n = 39). Data are fold change of mean ± SD relative to samples of WT; by Student’s *t* test; *p ≤ 0.05; **p ≤ 0.01; ***p ≤ 0.001.

Figure 3A shows that the relative mRNA expression of *Hmgcr* was moderately increased in 6-month-old (1.5-fold) and 12-month-old (1.3-fold) *Abcc6*^*−/−*^ mice compared to WT mice. The transcript level of *Fdft1* was significantly downregulated by 30% in the liver tissue of 12-month-old *Abcc6*^*−/−*^ mice, whereas younger *Abcc6*^*−/−*^ mice showed a 1.8-fold increase in *Fdft1* expression. Moreover, hepatic mRNA expression of *Lss* was significantly elevated up to 1.5-fold in 6-month-old *Abcc6*^*−/−*^ mice (Fig. 3A).

### Gene expression and serum levels of apos in Abcc6 deficiency

In comparison to WT mice, expression of *Apoa2* was significantly reduced in the liver samples of 6- and 12-month old *Abcc6*^*−/−*^ mice (Fig. 3B), while *Apoe* mRNA expression was 1.5-fold higher in the liver samples of 6-month old *Abcc6*^*−/−*^ mice. Furthermore, gene expression of *Apoa1* showed a 1.4-fold increase and the transcript level of *Apoc1* was 1.1-fold elevated in the liver tissue of 12-month old *Abcc6*^*−/−*^ mice (Fig. 3B). ApoC-II and ApoC-III mRNA expression levels were not different between *Abcc6*^*−/−*^ and WT (Suppl. Figure 1).

**Figure 4:**
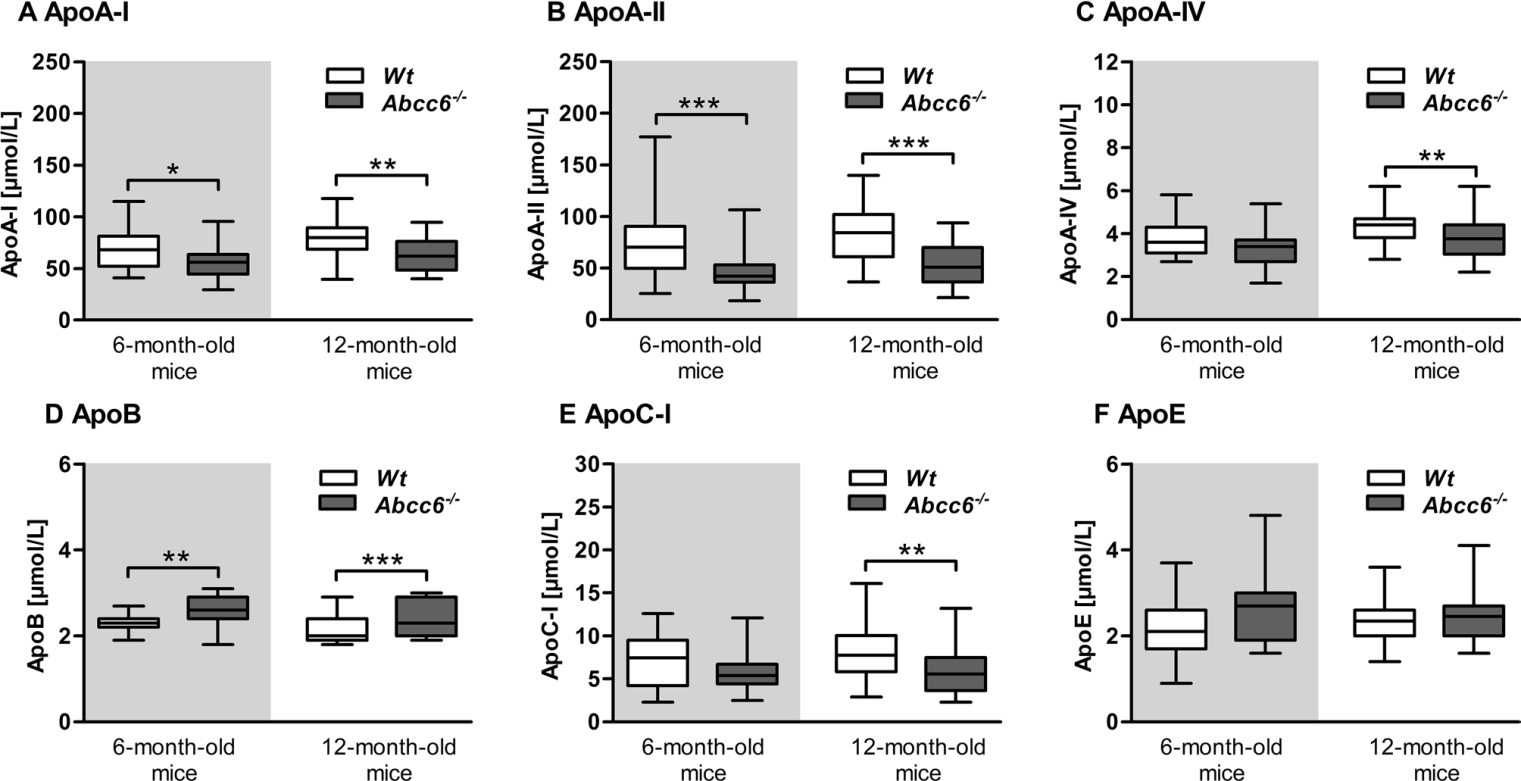
Reduced serum levels of HDL-associated apolipoproteins and elevated ApoB serum levels in Abcc6 knockout mice. Apolipoprotein serum levels of WT and *Abcc6*^*−/−*^ mice (6-month-old: WT n = 28, *Abcc6*^*−/−*^ n = 19; 12-month-old: WT n = 41, *Abcc6*^*−/−*^ n = 40). (**A**) ApoA-I, (**B**) ApoA-II, (**C**) ApoA-IV, (**D**) ApoB, (**E**) ApoC-I and (**F**) ApoE. Data are mean ± SD; one factorial variance analysis; *p ≤ 0.05; **p ≤ 0.01; ***p ≤ 0.001.

In addition to the gene expression analysis, we determined apo serum concentrations (Fig. 4A–D). The levels of ApoA-I were significantly decreased in both age classes of *Abcc6*^*−/−*^ mice compared to WT mice (6-month-old: − 18%; 12-month-old: − 18%, Fig. 4A). An even stronger reduction of Apo-II serum levels was demonstrated in *Abcc6*^*−/−*^ mice (6-month-old: − 36%; 12-month-old: − 34%, Fig. 4B). Twelve-month-old *Abcc6*^*−/−*^ mice also showed significantly lower ApoA-IV and ApoC-I levels (− 12% and − 25%, respectively, Fig. 4C,E). As opposed to these reductions, ApoB was significantly increased by 12% in both 6- and 12-month-old *Abcc6*^*−/−*^ mice (Fig. 4D). ApoE serum concentrations were not different between *Abcc6*^*−/−*^ and WT mice (Fig. 4F).

### Analysis of enzymes lipoprotein metabolizing in Abcc6-deficient mice

**Figure 5:**
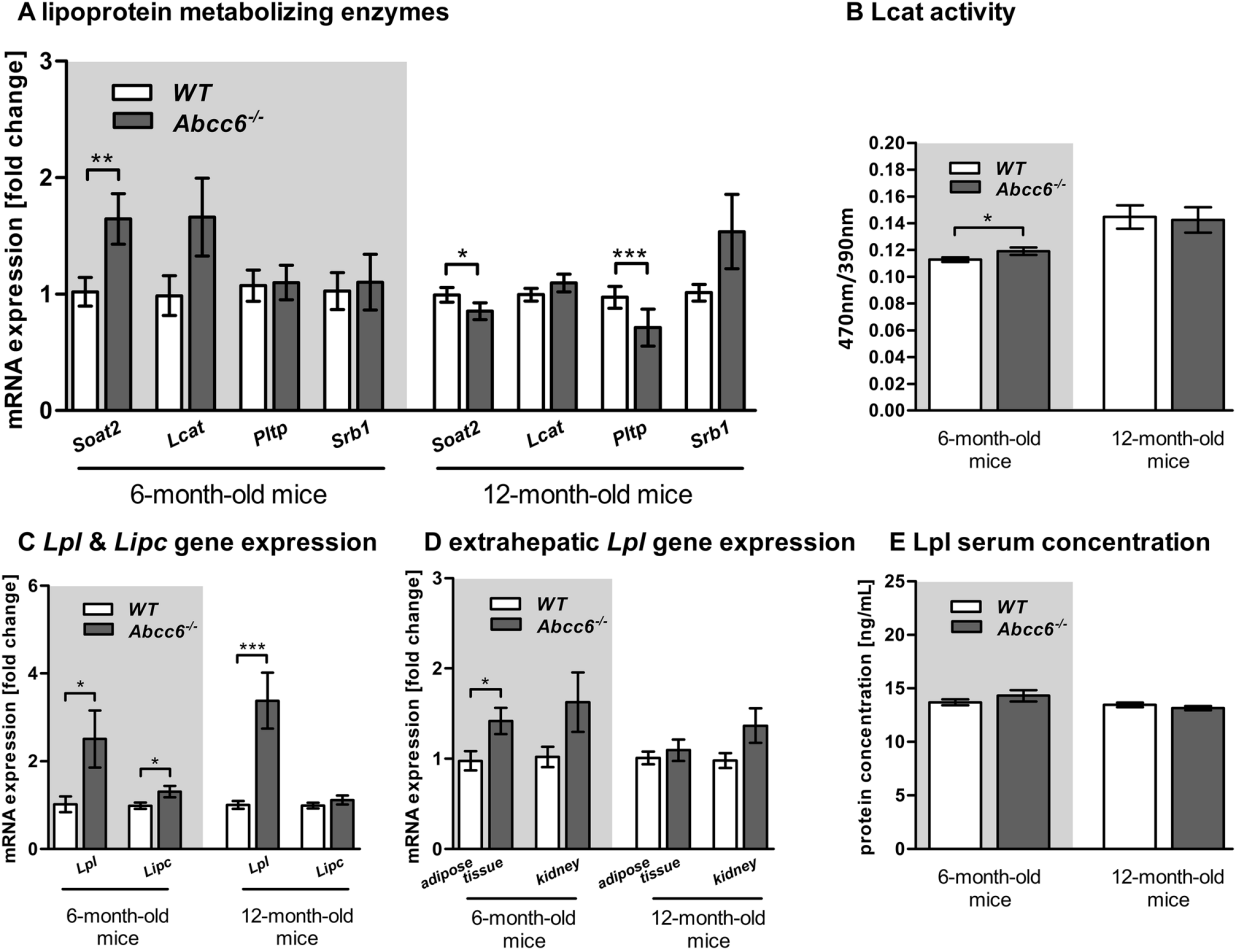
Expression alteration of lipoprotein metabolizing enzymes in Abcc6 knockout mice. (**A**) Relative hepatic gene expression levels of lipoprotein metabolizing proteins (sterol *O*-acyltransferase (*Soat2*), *Lcat*, phospholipid transfer protein (*Pltp*) and scavenger receptor B1 (*Srb1*)) of WT and *Abcc6*^*−/−*^ mice (6-month-old: WT n = 22, *Abcc6*^*−/−*^ n = 25; 12-month-old: WT n = 37, *Abcc6*^*−/−*^ n = 39). Data are fold change of mean ± SD relative to samples of WT. (**B**) The lecithin-cholesterol acyltransferase (Lcat) activity was determined in serum of WT and *Abcc6*^*−/−*^ mice (6-month-old: WT: n = 22, *Abcc6*^*−/−*^: n = 15; 12-month-old: WT: n = 30 *Abcc6*^*−/−*^: n = 34). Data are mean ± SD. (**C**) Relative hepatic gene expression level of lipoprotein lipase (*Lpl*) and hepatic lipase (*Lipc*) of WT and *Abcc6*^*−/−*^ mice (6-month-old: WT n = 22, *Abcc6*^*−/−*^ n = 25; 12-month-old: WT n = 37, *Abcc6*^*−/−*^ n = 39). Data are fold change of mean ± SD relative to samples of WT. (**D**) Relative gene expression level of *Lpl* in the kidney and white adipose tissue of WT and *Abcc6*^*−/−*^ mice (6-month-old: WT: adipose tissue n = 22, kidney n = 25, *Abcc6*^*−/−*^: adipose tissue n = 18, kidney n = 23; 12-month-old: WT: adipose tissue n = 33, kidney n = 34, *Abcc6*^*−/−*^: adipose tissue n = 38, kidney n = 42). Data are fold change of mean ± SD relative to samples of WT. (**E**) Quantification of Lpl concentration in serum of WT and *Abcc6*^*−/−*^ mice (6-month-old: WT: n = 23, *Abcc6*^*−/−*^: n = 21; 12-month-old: WT: n = 33 *Abcc6*^*−/−*^: n = 29). Data are mean ± SD; Student’s *t* test; *p ≤ 0.05; ***p ≤ 0.001.

In comparison to WT mice, higher transcript levels of sterol o-acyltransferase 2 (*Soat2*) (1.6-fold) were found in the liver tissue of 6-month-old *Abcc6*^*−/−*^ mice, whereas expression was repressed in older *Abcc6*^*−/−*^ mice (Fig. 5A). Furthermore, phospholipid transfer protein (*Pltp*) mRNA expression was reduced by 30% in the liver tissue of 12-month-old *Abcc6*^*−/−*^ mice. Phospholipase activity of Lcat was determined in a fluorometric assay in which lower signal intensity corresponds to higher enzyme activity. Figure 5B show that Lcat activity was lower by 5% in 6-month-old *Abcc6*^*−/−*^ mice compared to WT mice. However, differences in older mice were insignificant.

We observed significantly higher *Lpl* gene expression in the liver tissue of 6-month-old *Abcc6*^*−/−*^ mice (2.5-fold) than in WT mice. The expression of hepatic *Lpl* was also highly induced in 12-month-old *Abcc6*^*−/−*^ mice (3.4-fold; Fig. 5C). The extrahepatic expression level of *Lpl* was only significantly increased in the white adipose tissue of 6-month-old *Abcc6*^*−/−*^ mice (1.4-fold). The transcript level of hepatic lipase (*Lipc*) was significantly upregulated in 6-month-old *Abcc6*^*−/−*^ mice (1.3-fold; Fig. 5D). In contrast to the elevated *Lpl* gene expression in *Abcc6*^*−/−*^ mice, no differences of protein expression were found in these mice (Fig. 5E).

### Altered gene and protein expression of Pcsk9 and LDL receptor (Ldlr) in Abcc6-deficient mice

**Figure 6:**
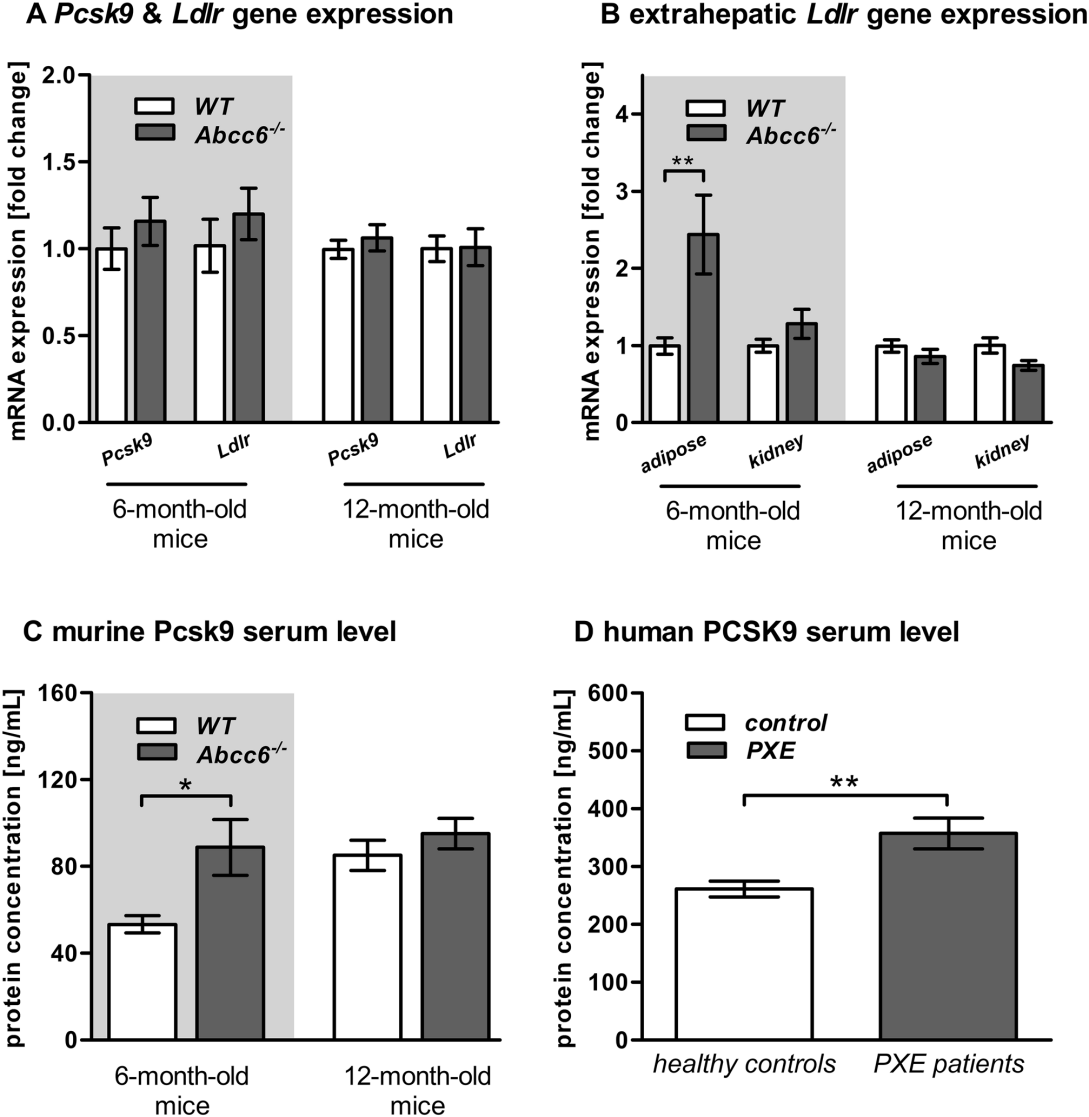
Altered gene and protein expression analysis of proprotein convertase subtilisin/kexin type 9 (Pcsk9) and low-density lipoprotein receptor (Ldlr) in Abcc6 knockout mice and PXE patients. (**A**) Relative hepatic gene expression level of proprotein convertase subtilisin/kexin type 9 (*Pcsk9*) and LDL receptor (*Ldlr*) of WT and *Abcc6*^*−/−*^ mice (6-month-old: WT n = 22, *Abcc6*^*−/−*^ n = 25; 12-month-old: WT n = 37, *Abcc6*^*−/−*^ n = 39). Data are fold change of mean ± SD relative to samples of WT. (**B**) Relative gene expression level of *Ldlr* in the kidney and white adipose tissue of WT and *Abcc6*^*−/−*^ mice (6-month-old: WT: adipose tissue n = 22, kidney n = 25, *Abcc6*^*−/−*^: adipose tissue n = 18, kidney n = 23; 12-month-old: WT: adipose tissue n = 33, kidney n = 34, *Abcc6*^*−/−*^: adipose tissue n = 38, kidney n = 42). Data are fold change of mean ± SD relative to samples of WT. (**C**) Quantification of Pcsk9 concentration in serum of WT and *Abcc6*^*−/−*^ mice (6-month-old: WT: n = 27, *Abcc6*^*−/−*^: n = 21; 12-month-old: WT: n = 35 *Abcc6*^*−/−*^: n = 31). Data are mean ± SD. (**D**) Quantification of PCSK9 concentration in serum of healthy controls and PXE patients (healthy controls: n = 36, PXE patients: n = 36). Data are mean ± SD; Student’s *t* test; *p ≤ 0.05; **p ≤ 0.01.

The expression of *Pcsk9* and *Ldlr* in the liver tissue of Abcc6-deficient and WT mice was similar (Fig. 6A). By contrast, the relative mRNA expression of *Ldlr* in the white adipose tissue of 6-month-old *Abcc6*^*−/−*^ mice was higher by factor 2.4 compared to WT mice (Fig. 6B).

The Pcsk9 concentration in serum of 6-month-old *Abcc6*^*−/−*^ mice was 67% higher than in WT mice (Fig. 6C). In line with this, PXE patients also had 41% higher PCSK9 serum concentrations than healthy controls (Fig. 6D).

## Discussion

Human genetic studies have demonstrated that diverse sequence variations of well-known genes, such as *LIPC*, *ABCA1* or even *ABCC6,* which cause PXE, are not disease-causing, but associated with decreased HDL cholesterol^6,7,20,21^. This association could be directly related to a higher cardiovascular risk in PXE patients. Studies of human and mice found an increased carotid intima media thickness^22–24^ due to ABCC6 deficiency, which correlates with higher risk for cardiovascular complications^25^. In current case reports it has been noticed that PXE patients suffer from degenerated and calcified elastic fibers in heart tissue as well stenosis in coronary arteries^26,27^. Furthermore, in a cardiac ischemia–reperfusion injury mice model could be demonstrated that the infarct size in heart tissue was increased in *Abcc6*^*−/−*^ mice^28^.

In line with a previous study by Gorgels et al.^8^, which reported lower total and HDL cholesterol in 8-month-old but not in the 2.5-month-old *Abcc6*^*−/−*^ mice, we also observed an age-dependent reduction of serum total (− 10%) and HDL cholesterol (− 20%) in 6-month-old *Abcc6*^*−/−*^ mice. These reductions increased with increasing age. Hence, *Abcc6*^*−/−*^ mice at an age of 12 months presented lower concentrations of total (− 20%) and HDL cholesterol (− 25%). Whereas Guo et al.^9^, showed an increase of total cholesterol and triglyceride levels in 3-month-old *Abcc6*^*−/−*^ mice. These similar studies on mice seem to give conflicting results which should be reasoned with different ages of analyzed animals as well with using two distinct *Abcc6* knockout models by different gene targeting strategies^8,9,29^. Several studies have demonstrated that a low HDL cholesterol level is a pivotal and independent factor from LDL cholesterol to facilitate coronary heart diseases^30^. The two most important structure elements of HDL particles are ApoA-I and ApoA-II^31^. The ApoA-I protein in conjunction with ABCA1 is responsible for the biogenesis of HDL by serving as a lipid acceptor for excess cholesterol^32^. Another protein ApoC-I regulates activities of various enzymes associated with HDL metabolism. It is responsible for the activation of LCAT and the inhibition of LIPC^33^. Moreover, ApoC-I has an important influence on ApoB-mediated VLDL uptake in the liver. An overexpression of human *ApoC1* in a murine model led to a massive increase of serum cholesterol and triglycerides by the accumulation of VLDL^34^. Unexpectedly, an *Apoc1* knockout in mice was not accompanied by a hypolipidemic phenotype, but rather by normal lipid concentrations in the serum under a normal chow diet^35^. In this study, we showed for the first time that an Abcc6 deficiency leads to a reduction of ApoA-I, ApoA-II, ApoA-IV and ApoC-I serum concentration in *Abcc6*^*−/−*^ mice. This observation might explain the decreased HDL cholesterol level of *Abcc6*^*−/−*^ mice and further indicates reduced HDL biogenesis. Another explanation for decreased HDL cholesterol might be altered hepatic expression of Abca1. However, we previously showed that hepatic Abca1 expression is not altered in *Abcc6*^*−/−*^ mice^36^. A recent study by Dergunov et al. 2020 showed that increased ApoA-I serum levels might be associated with higher ApoA-I release of HDL particles^37^. Released ApoA-I determines the increase of cholesterol efflux of macrophages to circulating HDL. Thus, low level of plasma HDL particles may be compensated by their increased potency for ApoA-I release. The authors suggested ApoA-I release as a new HDL functional property. This is probably not the case in HDL particles in Abcc6-deficient mice suggesting altered HDL functionality. The dysalphalipoproteinemia due to Abcc6-deficiency seen here might lead to a probably decreased flux of cholesteryl ester to the liver and consequently increased hepatic cholesterol synthesis.

In contrast to cholesterol, plant sterols cannot be synthesized by mammalian cells and, thus, must be absorbed with the diet^38^. Serum phytosterol levels, especially of campesterol and β-sitosterol, reflect intestinal cholesterol absorption^38^ and are furthermore, inversely related to serum precursor concentrations but correlate positively with HDL cholesterol and negatively with cholesterol biosynthesis activity^39,40^. Here, we reported for the first time significantly reduced serum concentrations of esterified and total phytosterols, such as campesterol and β-sitosterol in both 6- and 12-month-old *Abcc6*^*−/−*^ mice. These findings provide a first indication of lower cholesterol and plant sterol absorption due to Abcc6 deficiency. Perhaps the Abcc6 transporter might be partly responsible for functional sterol absorption, as it was demonstrated that the Abcc6 protein is expressed in several parts of the intestine, which are responsible for lipid absorption, particularly for cholesterol uptake^41–43^. Furthermore, Hosen et al. generated a ranking list for the most likely physiological substrates of ABCC6 by in silico docking analysis. Lipids and bile acids were identified particularly as potential ABCC6 substrates. Thereby, the plant sterol brassicasterol (rank 3), β-sitosterol as (rank 41) and stigmasterol as (rank 48) were highly ranked^5^.

The ratio of cholesterol precursors to the cholesterol level is used for activity analysis of cholesterol biosynthesis^39,44^. It is notable that the relative concentration of free desmosterol to cholesterol correlates positively with hepatic HMGCR activity, highlighting the rate-limiting step of cholesterol biosynthesis^45^. In addition, cholesterol precursor concentrations in serum are positively related to overall cholesterol biosynthesis and negatively related to cholesterol absorption and HDL cholesterol levels^40^. Here, we describe for the first time that the ratio of zy/dh/7dhc to cholesterol was significantly higher by up to 25% in both age groups of *Abcc6*^*−/−*^ mice. These data support our hypothesis of an increased cholesterol biosynthesis due to Abcc6 deficiency. However, the ratio of lanosterol to cholesterol was unchanged in 6-month-old *Abcc6*^*−/−*^ mice and significantly reduced in 12-month-old *Abcc6*^*−/−*^ mice. The precursor molecule lanosterol is able to induce the degradation of HMGCR protein^46^, thus, reduced lanosterol levels will further trigger cholesterol synthesis. We demonstrated in previous cell culture experiments that dermal fibroblasts from PXE patients have a significantly higher HMGCR mRNA expression and activity than healthy control cells^11^. Furthermore, Guo et al. observed in a previous study that a high dose atorvastatin treatment, a competitive HMGCR inhibitor, of *Abcc6*^*−/−*^ mice has a preventive effect on vibrissae calcification^9^. Statins may be able to reduce protein prenylation of G protein RhoA by the inhibition of HMGCR and related metabolic pathways, which activated the bone morphogenetic protein 2 (BMP2) signaling and, therefore, mineralization facilitates^10^. Furthermore, Hosen et al. found that BMP2 signaling in *Abcc6*^*−/−*^ mice are active at calcified sites, such as vibrissae and eyes. It was also demonstrated that this signaling pathway is upregulated in human fibroblasts from PXE patients in vitro^47^.

**Figure 7:**
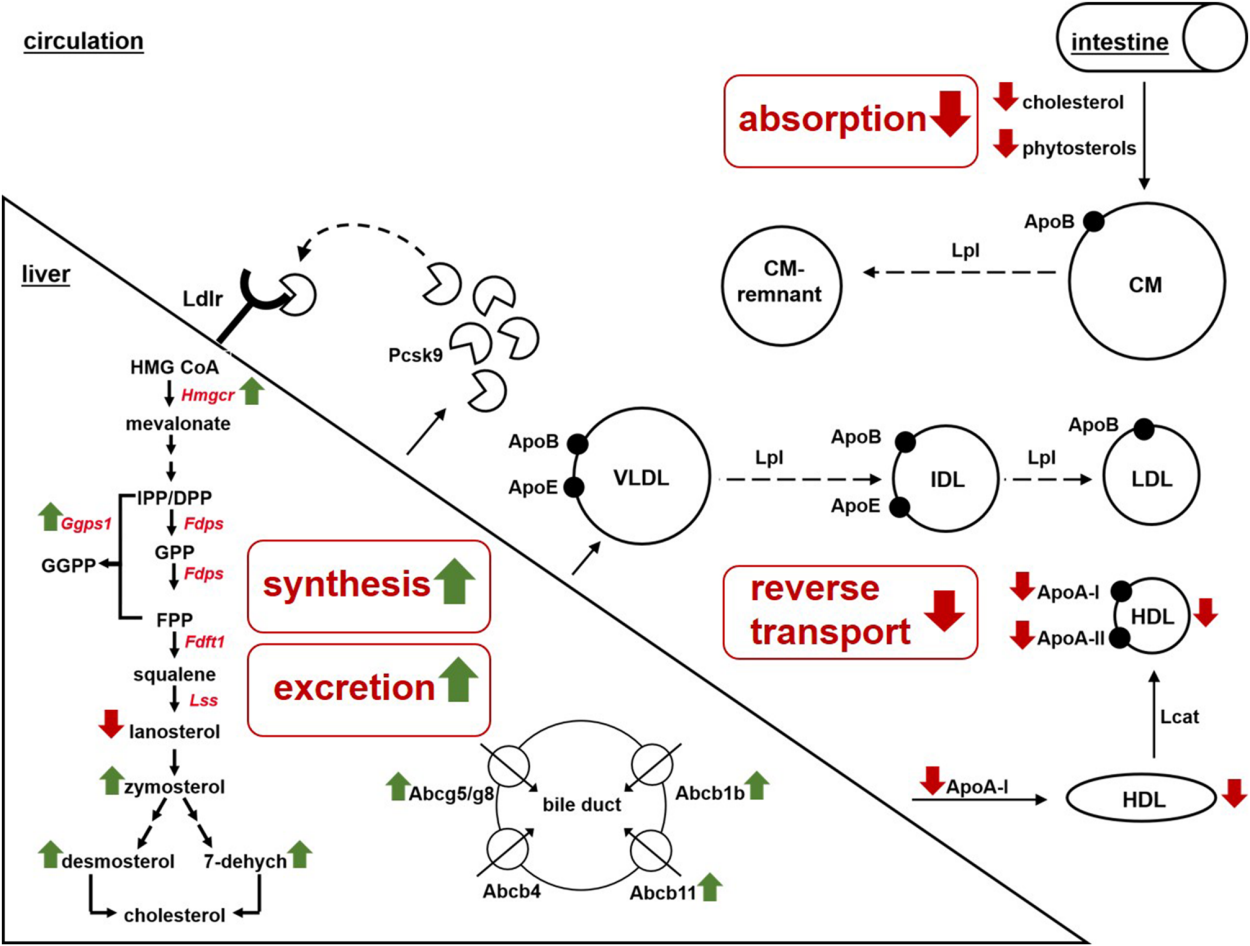
Hypothetic model of Abcc6 function in cholesterol homeostasis of mice. This model summarizes the results obtained in this study and illustrates the possible effects of an Abcc6 loss of function in maintaining cholesterol homeostasis in mice. We further demonstrated a disturbed reverse cholesterol transport by reduction of important apolipoproteins for HDL functionality in serum of *Abcc6*^*−/−*^ mice (ApoA-I and ApoA-II). We found elevated cholesterol precursors in serum of Abcc6 knockout mice (zymosterol, desmosterol, 7-dehydrocholesterol) as hint for an increased overall cholesterol synthesis. In a former study, we showed that Abcg5/Abcg8 expression in the liver of *Abcc6*^*−/−*^ mice was induced, suggesting an increased sterol excretion by the liver^50^. In summary, we propose that Abcc6-deficiency in mice might be accompanied by a reduced absorption of cholesterol and thereby phytosterols in the intestine. Green arrows indicate increased target expression, increased protein/metabolite concentration or activation of a pathway, red arrows indicate the repression of target expression or protein/metabolite concentration or inactivation of a pathway. See text for further details. *CM* chylomicron, *Lpl* lipoprotein lipase, *Pcsk9* proprotein convertase subtilisin/kexin type 9, *Ldlr* LDL receptor, *IDL* intermediate density lipoprotein, *Abc* ABC-transporter, *Fdps* farnesyl pyrophosphate synthase, *Fdft1* farnesyl diphosphate farnesyl transferase 1, *Ggps1* geranylgeranyl pyrophosphate synthase 1, *Lss* lanosterol synthase, *IPP/DPP* isopentenyl pyrophosphate/dimethyallyl pyrophosphate, *GPP* geranyl pyrophosphate, *GGPP* geranylgeranyl pyrophosphate, *FPP* farnesyl pyrophosphate, *7-dehydroch* 7-dehydrocholesterol.

We described here for the first time that Abcc6 deficiency in mice is linked to lower plant sterol and apolipoprotein levels and postulate a disturbed sterol absorption and HDL metabolism. Whereas Abcg5/Abcg8 own there physiological function in the biliary efflux of cholesterol metabolites, we propose a physiological function of ABCC6 in lipoprotein metabolism (e.g. packing of lipoprotein particles), probably influencing the content and subsequently the functional capacity of lipoprotein particles. Our results underline the role of Abcc6 in cholesterol homeostasis and indicate impaired cholesterol metabolism as an important pathomechanism involved in PXE manifestation (summarized in Fig. 7). These findings raise new questions about how cholesterol and lipid metabolism are regulated due to Abcc6 deficiency. Further studies are definitely necessary to understand the complex gene regulatory network and tissue specificity regulating cholesterol metabolism due to ABCC6 deficiency.

## Materials and methods

All experiments within this study were performed in accordance with relevant guidelines and regulations.

### Animals

All experiments within this study were performed using dead animals. Mice were killed solely for the use of their organs or tissues without incriminating experiments. Therefore, animal preparations comply with the law on animal welfare of Germany used for scientific purposes, an ethical approval for our study was not required. The *Abcc6*^*−/−*^ mice were generated on a hybrid background of C57BL/6 and 129/Ola and were backcrossed to C57BL/6 for more than 5 generations^8^. Mice were housed under standard conditions in a pathogen-free central animal facility of Bielefeld University (Germany) and kept with water and food (normal chow) ad libitum and therefore non-fasted blood samples were used for lipid analysis. In the present study, we used *Abcc6* (+/+) littermates and pure C57BL/6 mice as WT control mice. Mice (males and females), aged 6 months ± 2 weeks and 12 months ± 4 weeks, were anesthetized intraperitoneal with 0.65 mg ketamine, 0.02 mg acepromazine and 0.13 mg xylazine per 10 g bodyweight and sacrificed by bleeding subsequent to cervical dislocation. After opening the thorax, the right ventricle of the heart was cut for liver perfusion via the hepatic portal vein with PBS. Liver, kidneys and perigonadal white adipose tissue were collected, immediately frozen in liquid N_2_ and stored at − 80 °C until use. Serum was obtained by centrifugation of the blood samples collected at 3000*g* for 10 min at room temperature. Serum was stored at − 80 °C until use^36^.

### Patient characteristics

The diagnosis of PXE in all patients was consistent with the consensus criteria reported. Eleven male [mean ± SD age, 47.8 ± 5.5 years] and 25 female [38.7 ± 11.4 years] PXE patients, and 11 male [47.8 ± 5.5 years] and 25 female [38.7 ± 11.4 years] blood donors were included in this study as healthy controls. All patients and controls gave their written informed consent for participation in the study. The study was conducted in accordance with the Declaration of Helsinki and approved by the ethics Committee of the HDZ NRW, Department of Medicine, Ruhr University of Bochum (registry no. 32/08). Selected lipid parameters were measured and are shown in Supplementary Table 2.

### Serum levels of mouse pre-heparin Lpl

The concentration of pre-heparin Lpl in serum was measured via a commercial enzyme-linked immunosorbent assay *Mouse LPL ELISA Kit* (Biozol Diagnostica GmbH, Eching, Germany), according to the manufacturer’s protocol. Samples were diluted 1:20.

### Serum levels of human and mouse PCSK9

Serum levels of PCSK9 were measured using the Human PCSK9 Quantikine ELISA Kit or Mouse PCSK9 Quantikine ELISA Kit (R&D Systems, Inc., Minneapolis, MN, USA), according to the manufacturer’s instructions. Human serum was diluted 1:20 and murine serum 1:200.

### Quantification of HDL and LDL/VLDL cholesterol

The HDL and LDL/VLDL cholesterol levels of serum were measured using an HDL and LDL/VLDL Quantification Colorimetic/Fluorometic Kit (BioVision Incorporated, Milpitas, USA), according to the manufacturer’s instructions. After precipitation, 10 µL of the HDL and 20 µL of the LDL/VLDL suspension were used for photometric quantification.

### Quantification of oxLDL cholesterol

The oxLDL cholesterol levels of serum were determined by a commercial Mouse oxLDL ELISA Kit (Cloud-Clone Corp., Houston, USA), according to the manufacturer’s instructions. Samples were diluted 1:100.

### Lcat activity assay

The Lcat activity in serum was determined by using a commercial LCAT assay kit (Sigma-Aldrich, Taufkirchen, Germany; supplied by Roar Biomedical, Inc., New York, NY) according to the manufacturer’s protocol. An amount of 2 µL of undiluted serum were incubated with the intact substrate for 6 h at 37 °C.

### RNA extraction from tissue, cDNA synthesis and Quantitative Real-Time PCR

Total RNAs were extracted from 100 mg liver using QIAzol reagent (Qiagen, Hilden, Germany), followed by a purification using RNeasy Mini protocol (Qiagen, Hilden, Germany). Total RNA was treated with DNase I (Macherey-Nagel GmbH & Co. KG, Bottrop, Germany) on mini-columns to eliminate genomic DNA. The RNA quantification was assessed by using the NanoDrop 2000 spectrophotometer (Thermo Fisher, Schwerte, Germany) and RNA quality was determined using the Agilent RNA 6000 Nano Kit (Agilent Technologies, Ratingen, Germany), according to the manufacturer’s instructions. First-strand cDNA was synthesized from 1 µg of total RNA for each reaction using the SuperScript II Reverse Transcriptase Kit (Thermo Fisher, Schwerte, Germany), according to the manufacturer’s instructions. The cDNA was diluted 1:5 or 1:10 with water, depending on the target gene, and stored at − 20 °C prior to quantitative real-time PCR (qRT-PCR). The qRT-PCR was performed on a LightCycler480 (Roche, Mannheim, Germany) using Lightcycler480 MasterCycler SYBR Green (Roche, Mannheim, Germany) to assess the mRNA expression levels of target and reference genes. All intron-spanning primers used for qRT-PCR analysis were designed with Clone Manager Suite 7 (Scientific & Educational Software), synthesized by Biomers (Ulm, Germany) and are listed in the Supplementary Table 1. The PCR thermal cycling conditions contained an initial incubation of 5 min at 95 °C, followed by 45 cycles of 10 s denaturation at 95 °C, primer-specific annealing for 15 s at 65 °C or 59 °C, and 20 s elongation and detection of the amplicon at 72 °C. Finally, a melting curve analysis of the amplicon was performed. Each cDNA sample was run in technical triplicates. Water was used as a negative control for each primer pair. The relative amount of target mRNA in each sample was calculated using the ΔΔCt method, as previously described^48^. Relative mRNA expression levels were corrected by PCR efficiency and the reference genes normalization factor, by normalizing target mRNA Ct values to those of glyceraldehyde-3-phosphate dehydrogenase (*Gapdh*), hypoxanthine phosphor-ribosyltransferase 1 (*Hprt*) and beta-2 microglobulin (*ß2m*) (6-month-old mice) or *Gapdh, Hprt* and eukaryotic translation initiation factor 3 subunit A (*Eif3a*) (12-month-old mice). A cutoff for no detectable mRNA expression was set to a Ct value of 35^36^.

### Quantification of apos

Murine serum apos A-I, A-II, A-IV, B, C-I and E were quantified via tryptic proteotypic peptides applying liquid chromatography coupled to tandem mass spectrometry (LC–MS/MS). Method parameters including sample preparation were adapted from a standardized targeted proteomics assay for human apos^49^.

### Quantification of free and esterified sterols

Quantification of free and esterified cholesterol, cholesterol precursors, such as free lanosterol and free zymosterol/desmosterol/7-dehydrocholesterol (zy/de/7dhc), and phytosterols, such as brassicasterol, campesterol, β-sitosterol, and stigmasterol (mg/mL), were determined as published^50,51^.

### Statistical analysis

Data are presented as means with corresponding standard error of the means (SD). Graphic data processing and statistical analysis were performed with GraphPad Prism 5 (GraphPad Software, Inc., CA, USA), using Student’s *t* tests for two group comparisons (two-tailed) and the non-parametric Mann–Whitney *U* test for data which are not Gaussian distributed. Data were checked for normality using the Shapiro–Wilk test. From inhomogeneous variations a correction of the t-value according to Welch was carried out. In case of more than two groups the comparisons were analyzed using single factor variance analysis with Bonferroni correction. Statistical significance was accepted at p ≤ 0.05.

## Supplementary Information

Supplementary Information.
