# Supplementary material for: Genetic deletion of Abcc6 disturbs cholesterol homeostasis in mice
Source: Sci Rep. 2021 Jan 22;11:2137. doi: 10.1038/s41598-021-81573-1 (PMC7822913; doi:10.1038/s41598-021-81573-1)
Supplement: Supplementary file 1 — Supplementary Information. [file 41598_2021_81573_MOESM1_ESM.docx]

**Supplemental Data complementing the Manuscript**

**Genetic deletion of Abcc6 disturbs cholesterol homeostasis in mice**

Bettina Ibold (bibold@hdz-nrw.de)^1^, Janina Tiemann (jtiemann@hdz-nrw.de)^1^, Isabel Faust (ifaust@hdz-nrw.de)^1^, Uta Ceglarek (uta.ceglarek@medizin.uni-leipzig.de)^2^, Julia Dittrich (julia.dittrich@medizin.uni-leipzig.de)^2^, Theo G.M.F. Gorgels (theo.gorgels@mumc.nl)^3,4^, Arthur A.B. Bergen (aabergen@amc.uva.nl)^4,5^, Olivier Vanakker (olivier.vanakker@ugent.be)^6^, Matthias Van Gils (Matthias.vangils@ugent.be)^6^, Cornelius Knabbe (cknabbe@hdz-nrw.de)^1^, Doris Hendig (dhendig@hdz-nrw.de)^1^*

^1^Institut für Laboratoriums- und Transfusionsmedizin, Herz- und Diabeteszentrum Nordrhein-Westfalen, Universitätsklinik der Ruhr-Universität Bochum, 32545 Bad Oeynhausen, Germany

^2^Institut für Laboratoriumsmedizin, Klinische Chemie und Molekulare Diagnostik, Universitätsklinikum Leipzig, 04103 Leipzig, Germany

^3^University Eye Clinic Maastricht, Maastricht University Medical Center,

6202 AZ Maastricht, The Netherlands

^4^Netherlands Institute for Neurosciences (NIN-KNAW), Amsterdam, The Netherlands

^5^Academic Medical Centre, University of Amsterdam, 1100 DD Amsterdam, The Netherlands

^6^Center for Medical Genetics, Ghent University Hospital, 9000 Ghent, Belgium

* Corresponding author: Dr. Doris Hendig

Phone: ++49 (0)5731/97-3816 or 2003

Fax: ++49 (0)5731/97-2307

E-Mail: [dhendig@hdz-nrw.de](mailto:dhendig@hdz-nrw.de)

**Suppl. Figure 1:**

**
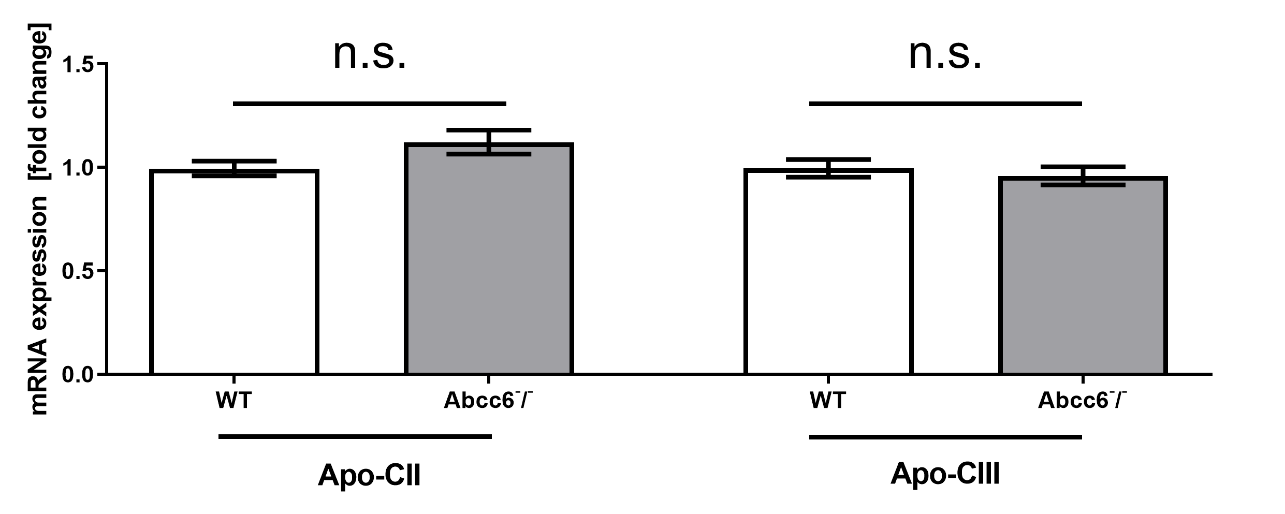
**

**Legend to**

**Supplemental Figure 1: Gene expression of apolipoproteins Apoc2 and Apoc3 in Abcc6 knockout mice.** apolipoproteins of WT and *Abcc6*^-/-^ mice, 12-month-old: WT n = 37, *Abcc6^-/-^* n = 39). Data are shown as mean ± SD relative to samples of WT; by Student’s t-test; n.s. not significant.

**Suppl. Table 1.**  Primer sequences used for qPCR

| **Target gene** | **Sequence 5´- 3´** | **Reference sequence^A^** | **Product size (bp)** | **Annealing (°C)^B^** |  |
| --- | --- | --- | --- | --- | --- |
| ***Actb***  *actin, beta* | TGCTGTCCCTGTATGCCTCT  AGGTCTTTACGGATGTCAACG | NM_007393.4 | 463 | 59 |  |
| ***Gapdh***  *glyceraldehyde-3-phosphate dehydrogenase* | GCATCTTGGGCTACACTGAGG  GGGTGGTCCAGGGTTTCTTAC | NM_008084.3 | 211 | 59 |  |
| ***ß2m***  *beta-2 microglobulin* | GGTCTTTCTGGTGCTTGTCTC  GCAGGCGTATGTATCAGTCTC | NM_009735.3 | 280 | 59 |  |
| ***Eif3a***  *eukaryotic translation initiation factor 3A* | GAGTATCAGGAGCGAGTCAAG  CCTCTCATCATCCCGAGTTTC | NM_010123.3 | 255 | 59 |  |
| ***Apoa1***  *apolipoprotein A-I* | AAGAAGAGCTGGACACCCAGAC  GCCCAAGGAGGAGGATTCAAAC | NM_009692.4 | 247 | 59 |  |
| ***Apoa2***  *apolipoprotein A-II* | GATATGCAGAGCCTGTTC  CTGGCACATCTCACTTAG | NM_013474.2 | 235 | 59 |  |
| ***Apoa4***  *apolipoprotein A-IV* | GTGTAGCCGAAACTGTCC  GAGCTGCTGAGTGACATC | NM_007468.2 | 213 | 59 |  |
| ***Apob***  *apolipoprotein B* | TGGCTCTGATCCCAAATCC  ACTTGAGGGCTGGCATTAC | NM_009693.2 | 279 | 65 |  |
| ***Apoc1***  *apolipoprotein C-I* | TTCATCGCTCTTCCTGTC  CGTGGTCTTCAACTTCTC | NM_007469.5 | 249 | 59 |  |
| ***Apoe***  *apolipoprotein E* | CGTGCTGTTGGTCACATTGC  TCCATCAGTGCCGTCAGTTC | NM_009696.4 | 207 | 59 |  |
| ***Hmgcr***  *3-hydroxy-3-methylglutaryl coenzyme A* *reductase* | AGGACTGTGAGGCTACAATG  GCGTTATCGTCAGGATGATG | NM_008255.2 | 222 | 59 |  |
| ***Fdps***  *farnesyl pyrohosphate synthase* | GCAGCCCTACTACCTGAAC  GAAGAACTCGCCCATCTCC | NM_134469.4 | 271 | 59 |  |
| ***Fdft1***  *farnesyl-diphosphate farnesyltransferase 1* | CATGCCTGCCGTCAAAGCTATC  ATCCTTTCCTCTGCCCAGTTCC | NM_010191.3 | 410 | 59 |  |
| **Target gene** | **Sequence 5´- 3´** | **Reference sequence^A^** | **Product size (bp)** | **Annealing (°C)^B^** |  |
| ***Ggps1***  *geranylgeranyl pyrophosphate synthase 1* | AGATCATCGTGGAACCGTCAG  TCCGGGTGATCAAGGGTTAAG | NM_010282.2 | 368 | 65 |  |
| ***Lss***  *lanosterol synthase* | CGAGTTCCTGCGGCTTTCAC  TCTGCTGCCCTGTAGTCTGG | NM_146006.2 | 420 | 59 |  |
| ***Soat2***  *sterol O-acyltransferase 2* | GAGCGACAAGATGAATGC  ATGCTGGAACCGAATAGG | NM_009338.3 | 266 | 59 |  |
| ***Lcat***  *lecithin-cholesterol transferase* | CACCATCTGGCTGGATTTCAAC  AAGCGGCATACATCTCCTCTAC | NM_008490.2 | 338 | 59 |  |
| ***Pltp***  *phospholipid transfer protein* | CAGACGTGTACGGCGCAAAG  ACACCGTCCCAGCATGGTAG | NM_011125.2 | 429 | 59 |  |
| ***Srb1***  *scavenger receptor B1* | GAGAACCGCAGCCTCCATTTCC  GCCCGTGAAGACAGTGAAGACC | NM_016741.2 | 330 | 59 |  |
| ***Lipc***  *hepatic lipase* | CGGGAAGAACAAGATTGG  TGATGGTCTGGGTTATGG | NM_008280.2 | 281 | 59 |  |
| ***Pcsk9***  *proprotein convertase subtilisin/ kexin type 9* | TGGTGCTGATGGAGGAGAC  GCTCTGGGCGAAGACAAAG | NM_153565.2 | 221 | 59 |  |
| ***Lpl***  *lipoprotein lipase* | GTCGCCTTTCTCCTGATG  CTCTTGGCTCTGACCTTG | NM_008509.2 | 385 | 59 |  |
| ***Ldlr***  *low-density lipoprotein receptor* | TTCCAGTGGCCGTCTCTATTG  TCACATCTGAACCCGTGAGTC | NM_010700.3 | 209 | 59 |  |

A Reference sequence taken from GenBank. Accession numbers are presented (2017).

B Annealing temperature in °C.

**Suppl. Table 2**

**Biochemical lipid parameters of PXE patients.**

| Parameter^a^ | PXE patients | Normal range |
| --- | --- | --- |
| Cholesterol [mg/dl] | 201 ± 40.2 (125 - 284) | 110 - 220 |
| LDL [mg/dl] | 82 ± 23 (41 - 137) | 0 – 150 |
| HDL [mg/dl] | 43 ± 9.5 (11 - 65) | 35 - 55 |

^a^Mean value ± SD, value range is shown within parentheses.

The diagnosis of PXE in all patients was consistent with the consensus criteria reported. PXE patients bear skin, eye and cardiovascular manifestation typical for PXE, mutational analysis of *ABCC6* completed PXE diagnostics. No phenotype scoring was performed. Cholesterol, LDL and HDL levels were measured with the clinical chemistry automate Architect c8000 from Abbott Diagnostics. Established normal reference values were used for evaluating lipid parameters of PXE patients. The measured mean value determined for lipid parameters cholesterol, HDL and LDL in PXE patients samples was within the normal reference change.
